# Microbial communities associated with the anthropogenic, highly alkaline environment of a saline soda lime, Poland

**DOI:** 10.1007/s10482-017-0866-y

**Published:** 2017-04-05

**Authors:** Agnieszka Kalwasińska, Tamás Felföldi, Attila Szabó, Edyta Deja-Sikora, Przemysław Kosobucki, Maciej Walczak

**Affiliations:** 1 Department of Environmental Microbiology and Biotechnology, Faculty of Biology and Environmental Protection, Nicolaus Copernicus University, Toruń, Poland; 2 Chair of Environmental Chemistry and Bioanalytics, Faculty of Chemistry, Nicolaus Copernicus University, Toruń, Poland; 3 Department of Microbiology, Eötvös Loránd University, Budapest, Hungary

**Keywords:** Alkaliphiles, Halophiles, Saline soda (Solvay) lime, 16S rRNA gene pyrosequencing

## Abstract

Soda lime is a by-product of the Solvay soda process for the production of sodium carbonate from limestone and sodium chloride. Due to a high salt concentration and alkaline pH, the lime is considered as a potential habitat of haloalkaliphilic and haloalkalitolerant microbial communities. This artificial and unique environment is nutrient-poor and devoid of vegetation, due in part to semi-arid, saline and alkaline conditions. Samples taken from the surface layer of the lime and from the depth of 2 m (both having pH ~11 and EC_e_ up to 423 dS m^−1^) were investigated using culture-based (culturing on alkaline medium) and culture-independent microbiological approaches (microscopic analyses and pyrosequencing). A surprisingly diverse bacterial community was discovered in this highly saline, alkaline and nutrient-poor environment, with the bacterial phyla Proteobacteria (representing 52.8% of the total bacterial community) and Firmicutes (16.6%) showing dominance. Compared to the surface layer, higher bacterial abundance and diversity values were detected in the deep zone, where more stable environmental conditions may occur. The surface layer was dominated by members of the genera *Phenylobacterium*, *Chelativorans* and *Skermanella*, while in the interior layer the genus *Fictibacillus* was dominant. The culturable aerobic, haloalkaliphilic bacteria strains isolated in this study belonged mostly to the genus *Bacillus* and were closely related to the species *Bacillus pseudofirmus*, *B. cereus*, *B. plakortidis*, *B. thuringensis* and *B. pumilus*.

**Electronic supplementary material:**

The online version of this article (doi:10.1007/s10482-017-0866-y) contains supplementary material, which is available to authorized users.

## Introduction

**Fig. 1:**
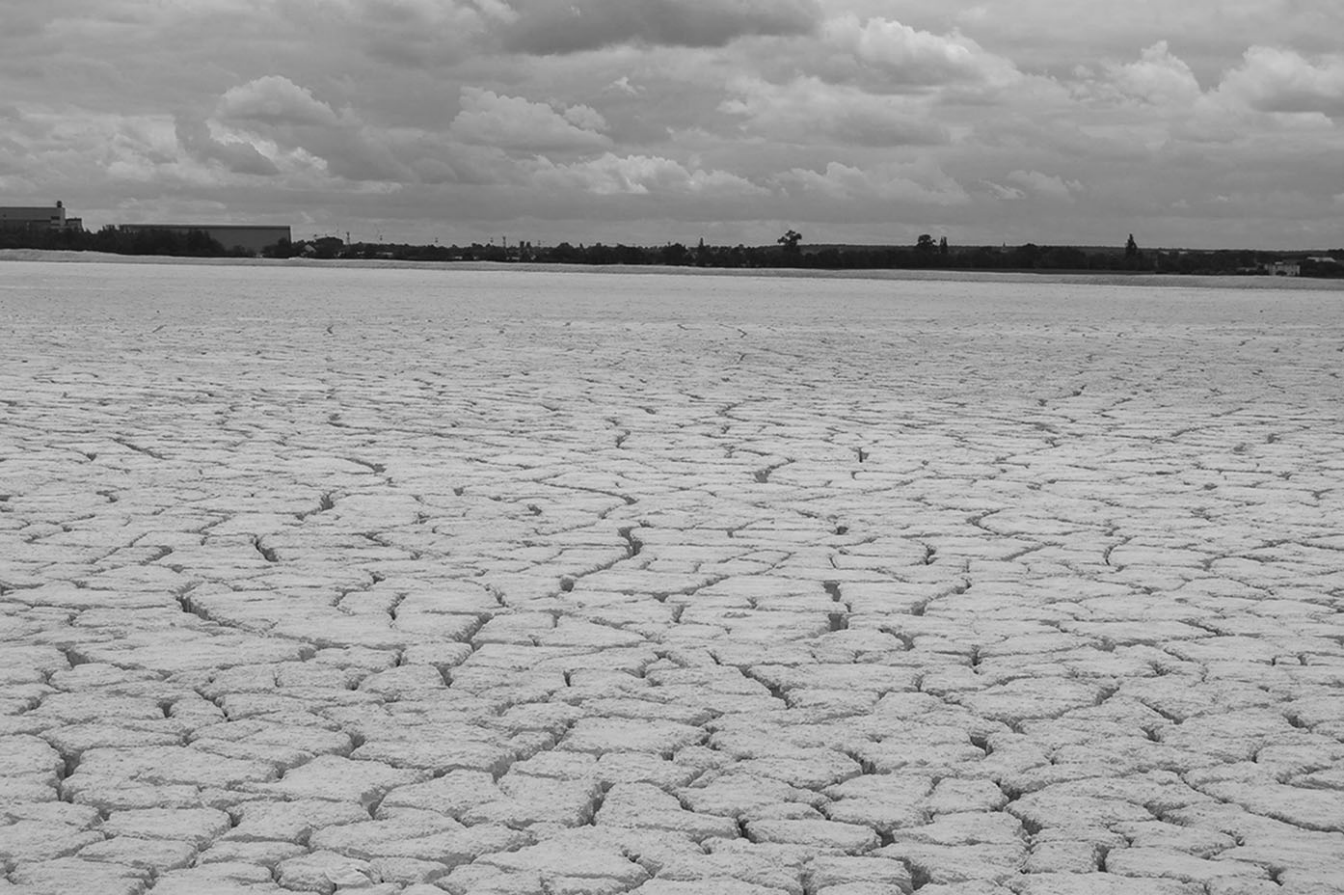
The surface of the saline soda lime deposited in the repository in Janikowo

Saline soda lime is a by-product of the Solvay soda process for the production of sodium carbonate from limestone and sodium chloride. The multistage Solvay process comprises: (I) sodium bicarbonate precipitation from brine saturated with ammonia and carbon dioxide, (II) conversion of sodium bicarbonate into the final product–sodium carbonate, and (III) regeneration of ammonia from ammonium chloride solution. Alkaline soda lime is generated as a by-product of the third stage of process. It has the form of a thick sludge and consists mainly of calcium carbonate (constitutes over 90% of the lime), amorphous calcium hydroxide (represents around 6%), and sodium and calcium chlorides (Ziółkowska et al. 2013). The remaining constituents include silicon dioxide and magnesium and aluminium salts. Since soda production started in Janikowo (Poland) in 1957, the alkaline lime has been deposited in the repository ponds called the “white seas” (Fig. 1). This unique, artificial environment is a potential habitat of haloalkaliphilic and haloalkalitolerant microorganisms due to high pH (~11) and high salt concentration (up to 423 dS m^−1^). It can be expected that these environmental conditions favour the selection of microorganisms capable of growing both in the presence of high salt concentrations and in conditions with highly alkaline pH (Ma et al. 2010).

Microbiomes of the natural saline-alkaline environments have been thoroughly investigated in recent years (Qazi 2013). However, studies have been focused mainly on microorganisms of soda lakes (Duckworth et al. 2000; Pikuta et al. 2003; Sorokin et al. 2011; Borsodi et al. 2008), while the microbial communities inhabiting natural and man-made soda (solonchak) soils and deserts have not been thoroughly studied (Sorokin et al. 2008; Pan et al. 2010). Microorganisms of unique and underexplored terrestrial saline and alkaline habitats require more consideration as knowledge of them is really limited. They live under adverse and unstable environmental conditions. Apart from extremely high concentrations of salt and alkaline pH, the microbes are exposed to seasonal or constant drought, excessively high or low temperatures, and oligotrophy (Pan et al. 2010). It is to be expected that haloalkaliphilic taxa predominating in dry or semi-arid land habitats would be similar to those previously found in soda lakes (Sorokin et al. 2008); however, there is still little evidence for complex microbial communities functioning under such harsh conditions.

Saline soda lime (an artificial, man-made environment) was chosen by us as a model of a nutrient-poor, semi-arid (annual precipitation less than 500 mm, Kędziora 2011), saline-alkaline habitat, resembling soda soils in such parameters like alkalinity, solidity, dryness and low nutrient content. The surface of the lime is permanently devoid of vegetation. It loses water seasonally due to strong evaporation and becomes hard and cracked (Fig. 1).

The aim of our study was to determine the abundance, biodiversity and community composition of bacteria inhabiting saline soda lime and to compare the communities of this unique and extreme environment with the microbiota inhabiting natural saline and alkaline environments. Two layers of the lime, from the surface and from a depth of two meters, corresponding to environments 1 and 10 years old, respectively, and differing in physicochemical properties were sampled and analysed.

## Materials and methods

### Study area

Saline soda lime has been deposited in Janikowo (Kuyavia, Central Poland; 52°46′45.0″N 18°07′30.0″E) since 1957. The repository of the lime covers an area of 200 ha, has the capacity of over 13,000,000 Mg and is composed of several ponds, which are separated by causeways. This area rises up to 16 m above the ground level (Regional Inspectorate for Environmental Protection 2013). Most of the ponds contain desalted lime (with chlorides concentration around 30 g kg^−1^), which is used in agriculture for de-acidification of soil. The particular pond from which samples were taken contains increased concentration of salts due to the deposition of lime sludge after cleaning a technological line used in wet and dry vacuum salt production.

### Saline soda lime samples

Samples of the saline soda lime were collected in September 2013 from the surface layer of the deposit (depth of 0–20 cm) and from a depth of 1.8–2.0 m using a telescopic Edelman mud auger (GeomorTechnik, Szczecin, Poland). The alkaline lime, a product of chemical processes, is more homogenous than soil. Therefore only ten samples were taken: five from the surface layer and five samples from the deep layer. The surface layer was the most recent, compared to the bottom layer, and the age of the two layers was estimated at 1 and 10 years, respectively. For each sampled layer, five separate subsamples were taken in the corners and in the centre of a 2.5 m square using a hand coring device (4 cm in diameter; Pepper and Gerba 2014). Samples (50 cm^3^) were placed in sterile glass jars and they were transported to the laboratory in a portable ice bag at 4 °C. In the laboratory, subsamples (10 g) from a particular layer were mixed thoroughly for subsequent microbial and physicochemical tests. All analyses started within 4 h after sample collection.

### Chemical analyses of soda lime

As a pre-treatment, solid and dried samples were hammered to reduce agglomeration and then homogenized in a roller mill. Gravimetric moisture of the saline soda lime was determined by the method of mass loss in drying. To avoid the known problem of imprecise pH measurement due to high salinity, the pH of the samples was determined by the alkacymetric titration of the aqueous lime suspensions (using 0.1:100 lime: solvent ratio, w/v). The suspensions were titrated until the indicator dye was stable (Ziółkowska et al. 2013). Electrical conductivity (EC) in extracts of the water-saturated lime paste was measured by the conductometric method. The concentration of chloride ions was determined by the argentometric titration with silver nitrate. Calcium and sodium ion concentrations were analysed with BWB XP (Halstead, England) flame photometer. Total organic carbon (TOC) was measured in a TOC 5000 analyzer combined with a SSM-5000A module Shimadzu (Kyoto, Japan). TOC values were calculated by subtracting inorganic carbon concentration (IC) from total carbon concentration (TC). Organic nitrogen concentration was calculated by subtracting ammonia nitrogen from total Kjeldahl nitrogen (TKN). TKN was determined in a Turbotherm system (Gerhardt, Konigswinter, Germany). Ammonia nitrogen concentration was measured using the Nessler method (Spectroquant Merck SQ 118, Darmstadt, Germany) after distillation in a Vapodest 20 system (Gerhardt, Konigswinter, Germany). Nitrate concentration was determined using the spectrophotometric method (Spectroquant Merck SQ 118, Darmstadt, Germany) after extracting nitrate from the sample with 2.0 M KCl. Total phosphorus content was measured with the spectrophotometric molybdenum blue method (Spectroquant Merck SQ 118, Darmstadt, Germany) after sample digestion with aqua regia. All analyses were performed in triplicate; the mean values with standard deviations (SD) are given in Table 1.

**Table 1:** Chemical properties of the saline soda lime

	Surface	Interior
pH	10.81 ± 0.10	10.84 ± 0.06
EC_e_ (dS m^−1^)	378 ± 20.20	423 ± 13.00
Chlorides (g kg^−1^)	108.64 ± 3.10	122.02 ± 2.36
Sodium (g kg^−1^)	56.22 ± 3.31	63.21 ± 2.84
Calcium (g kg^−1^)	23.97 ± 0.25	24.28 ± 0.18
Water content (%)	27.80 ± 1.39	68.85 ± 4.68
Total organic carbon (g kg^−1^)	2.94 ± 0.96	3.23 ± 0.82
Organic nitrogen (mg kg^−1^)	<LOD	32.97 ± 5.24
Nitrates (mg kg^−1^)	<LOD	<LOD
Ammonia nitrogen (mg kg^−1^)	<LOD	3.9 ± 0.20
Total phosphorus (mg kg^−1^)	29.63 ± 6.32	27.63 ± 5.98

The values are given as means ± SD; for organic nitrogen and ammonia nitrogen LOD (limit of detection) <0.01 mg kg^−1^, for nitrates LOD <0.001 mg kg^−1^

### Isolation and enumeration of bacteria

To isolate culturable bacteria and to estimate their number, 10 g of lime sample was added to 90 ml of NaCl solution (a concentration corresponding to the chloride content in a given sample). Mixtures were blended thoroughly, tenfold serial dilutions were prepared, and 0.1 ml of each suspension was spread onto three replicate plates. An isolation medium consisted of the lime extract (100 g dissolved in 1 l of distilled water, mixed for 20 min, filtered and adjusted to pH 10 using the buffer solution (Na_2_CO_3_–K_2_HPO_4_) according to Ntougias et al. (2006), enriched with glucose (0.5 g l^−1^) and yeast extract (0.2 g l^−1^), and solidified with agar (1.5%). The cultivation was performed in three parallel repetitions. All plates were incubated up to 21 days at 25° C. The number of bacteria was expressed as colony forming units (CFU) per g dry weight of the saline soda lime.

Isolates differing in appearance, colour, size and shape of bacterial colonies on the medium were chosen for subsequent taxonomic identification. Colonies were subcultured in a medium containing glucose (5 g l^−1^), yeast extract (1 g l^−1^), MgSO_4_ anhydrous (0.01 g l^−1^), and NaCl (60 g l^−1^), supplied with the buffer as given above. Then glycerol stocks of isolates were prepared and stored at −80 °C.

The total number of bacteria in alkaline lime samples was determined by the acridine orange direct count technique. 1 g of the lime (fixed with formaldehyde in 3.8% v/v final concentration, sterilized with MF-Millipore Membrane Filters, 0.22 µm) was placed into glass tubes containing 9 ml of filter-sterilized citric acid solution (final concentration 0.6 M). Citric acid was added in order to dissolve calcium carbonate present in the samples. For each suspension, three subsamples were stained with acridine orange (final concentration 0.01%) for 3 min and then the slides were immediately viewed under a microscope (Nicon Eclipse E200, Japan).

### Identification of bacterial strains based on the 16S rRNA gene

Total genomic DNA was extracted from the strains using the Gene MATRIX Bacterial & Yeast Genomic DNA Purification Kit (EURx, Gdańsk, Poland) following the instructions given by the manufacturer. The 16S rRNA gene was amplified by the PCR in a reaction mixture containing 1 U *Taq*DNA polymerase (EURx), 0.2 mM dNTP mixture (EURx), 1× Polbuffer B with 1.5 mM MgCl_2_ (EURx), 0.25 µM of universal primers 27F (5′-AGA GTT TGA TCM TGG CTC AG-3′, (Lane 1991)) and 1492R (5′-TAC GGY TAC CTT GTT ACG ACT T-3′ (Polz and Cavanaugh 1998)), and 1 µl of genomic DNA in a total volume of 20 µl. The thermal profile consisted of an initial denaturation at 95 °C for 3 min, 35 amplification cycles (denaturation at 95 °C for 30 s, annealing at 53 °C for 20 s and extension at 72 °C for 1 min 40 s) and a final extension at 72 °C for 5 min. PCR amplicons were checked on a 1 w/v  % agarose gel stained with Midori Green DNA Stain (Nippon Genetics Europe GmbH, Dueren, Germany). Sequencing reactions of PCR products were performed with primer 27F, using a Big Dye Terminator v 3.1 Cycle Sequencing Kit (Applied Biosystems, Thermo Fisher Scientific, Waltham, MA, USA) following the instructions given by the manufacturer. Capillary electrophoresis was performed by the Oligo sequencing laboratory (IBB PAS, Warsaw, Poland). Manual correction of automatic base calling on chromatograms was carried out using MEGA 6.0 software (Tamura et al. 2013). Nucleotide sequences of the bacterial strains obtained in this study were deposited to GenBank under the accession numbers KT387570-KT387597.

The taxonomic affiliation of bacterial strains was determined using the EzTaxon online tool (Kim et al. 2012). Additional closest relatives of isolates were identified with BLASTN and these sequences were also used in the phylogenetic analysis. Sequence alignment was performed with the SINA Aligner (Pruesse et al. 2012). Phylogenetic analysis including the search for the best-fit model was conducted with the MEGA 6.0 software (Tamura et al. 2013).

### Pyrosequencing analysis of the total bacterial community

Environmental genomic DNA was isolated from samples homogenized as follows: 10 g of sample was added to 90 ml citric acid (final concentration 0.6 M) in order to dissolve calcium carbonate, the suspension was centrifuged (Rotina, Hettich, Germany 4000×*g*, 20 C, 10 min), and washed twice with pH 8 phosphate buffer.

DNA extraction, PCR amplification, pyrosequencing and subsequent sequence analysis was performed as generally described by Felföldi et al. (2015). Bacteria-specific primers S-D-Bact-0341-b-S-17 and S-D-Bact-0785-a-A-21 (Klindworth et al. 2013) were applied for the triplicate amplification of the 16S rRNA gene using 0.4 U Phusion High Fidelity DNA polymerase (Thermo Fisher Scientific), 1× Phusion HF Buffer (Thermo Fisher Scientific), 0.2 mM dNTP (Fermentas, Vilnius, Lithuania), 0.4 µg/µl BSA (Fermentas), 0.5 µM of both primers and 1.0 µl of genomic DNA in a final volume of 20 µl, applying the thermal profile of initial denaturation at 98 °C for 5 min, 25 cycles (with denaturation at 95 °C for 40 s, annealing at 55 °C for 2 min and extension at 72 °C for 1 min) and final extension at 72 °C for 10 min. Sequencing was performed with the GS Junior platform (Roche, Basel, Switzerland). Data were processed with mothur v 1.33 (Schloss et al. 2009); briefly, sequences were quality filtered, denoised, singletons and putative chimeras were removed; and subsequent taxon identification was performed with the SINA aligner tool (Pruesse et al. 2012) using the ARB-SILVA SSU NR reference database–SILVA Release 119 (Quast et al. 2013). After the removal of archaeal, chloroplast and mitochondrial sequences from the dataset, operational taxonomic units (OTUs) were assigned at 97% similarity level, representing bacterial species. The number of obtained sequences was normalized to the sample having the lowest sequence count prior to the calculation of richness estimators and diversity indices in mothur. Association networks of major bacterial OTUs were constructed with Cytoscape 3.2.1 (Shannon et al. 2003) based on the 50 OTUs having the highest abundance in the total sequencing dataset.

Since plastid 16S rRNA gene references are underrepresented in the ARB-SILVA database, chloroplast sequences were analysed separately: the closest related sequences were retrieved from GenBank using BLASTN, and following an alignment with SINA, the subsequent phylogenetic analysis was performed with MEGA 6.0 (Tamura et al. 2013).

The NCBI Sequence Read Archive accession codes for the unique sequences obtained with pyrosequencing are SRS1279117 and SRS1279119.

## Results

### Lime characteristics

**Fig. 2:**
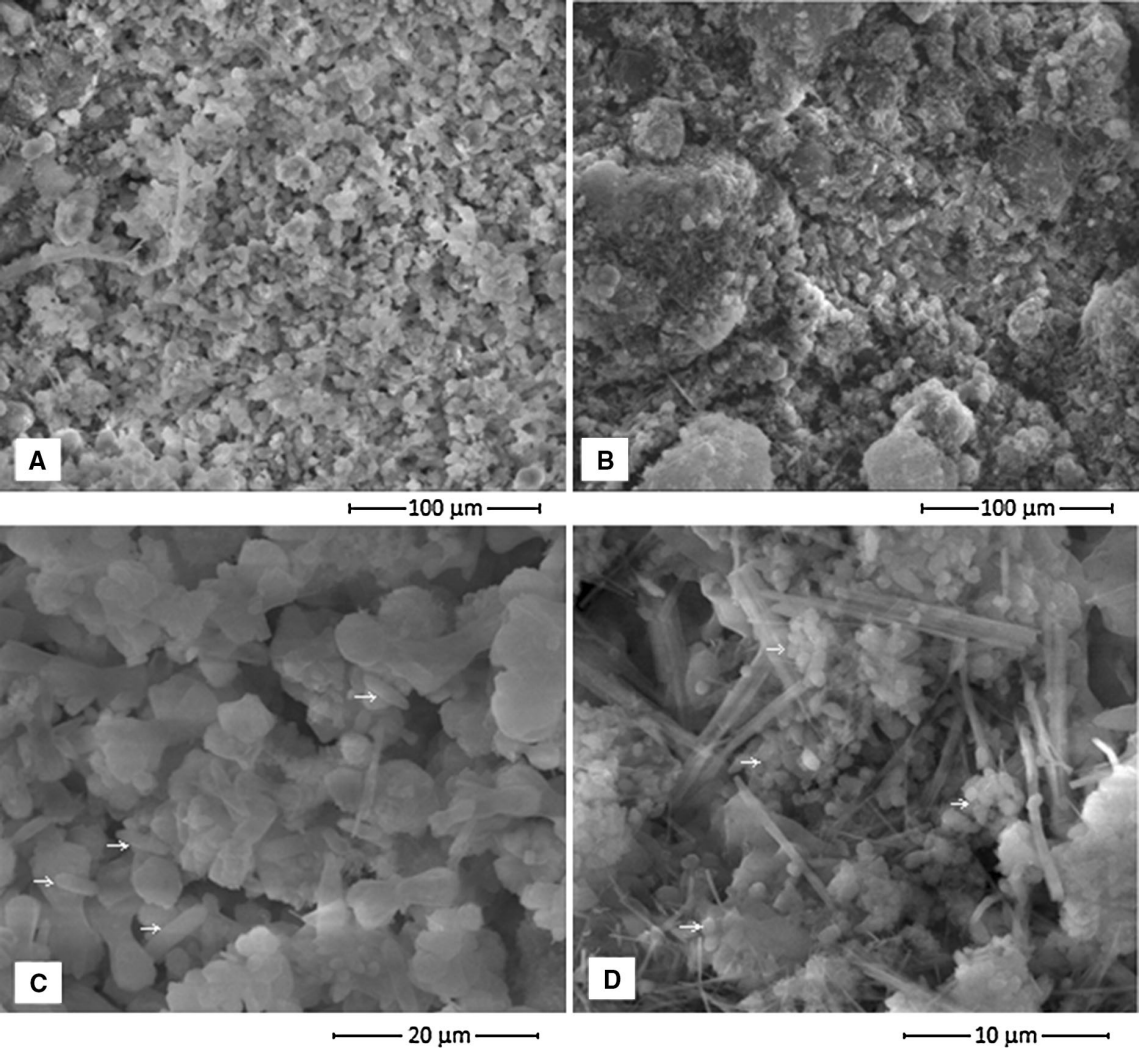
Physical structure of the saline soda lime **a** less compact surface layer, **b** more compact internal layer, **c**, **d** *arrows* point putative bacterial cells, covered with salts)

The physicochemical characteristics of the saline soda lime samples are presented in Table 1. The surface layer of alkaline lime and the layer from the depth of 2.0 m had similar alkaline pH, measured in a water suspension. The EC in saturated lime extracts was very high, up to 423 dS m^−1^. The water content was significantly higher in the deep layer of the lime compared to the surface layer. The samples contained a low concentration of nitrogen and organic carbon. Ammonia nitrogen was only detected at a depth of 2.0 m, and nitrates were not detected. In contrast to this, total phosphorus concentrations were rather high. The interior samples of the lime (Fig. 2b) were more compact compared to the surface layer (Fig. 2a).

### Total number of bacteria and number of culturable bacteria

The total number of bacteria determined by direct cell counting was much lower in the surface layer than in the lime at a depth of 2.0 m (Table 2). A similar trend was observed in the number of culturable bacteria. The number of colony forming units was one order of magnitude lower in the surface layer as compared with the deep layer. Both the direct count of bacteria and the number of culturable bacteria in the surface and the interior layer of saline-alkaline lime, differed significantly (p < 0.01).

**Table 2:** Abundance of microorganisms in the saline soda lime

	Surface	Interior
Culturable bacteria (CFUg^−1^ dry mass, 10^3^)	1.44 ± 0.12	34.82 ± 3.30
Total number of bacteria (cells g^−1^ dry mass, 10^6^)	7.52 ± 2.03	245.20 ± 95.55

The values are given as means (n = 3) ± SD

### Community structure

In total, 10,702 high-quality 16S rRNA gene sequence reads obtained by pyrosequencing were clustered at the 97% similarity level, resulting in 422 operational taxonomic units (corresponding to the bacterial species; Table 3), out of which 82 OTUs (19.4%) were shared among the samples. Compared to the deep layer, the rarefaction curve of the surface layer reached a higher level of saturation (Supplementary Fig. S1). The two datasets shared only a 0.177 Bray-Curtis similarity based on the abundance of the bacterial OTUs, which showed that the bacterial communities in the two layers were highly dissimilar.

**Table 3:** Pyrosequencing data statistics of the saline soda lime samples

Sample	Total	Surface	Interior
Number of sequences after quality filtration	10702	4263	6439
Bacterial sequences	6894	2150	4744
Archaeal sequences	1217	70	1147
Chloroplast sequences	2471	1959	512
Mitochondrial sequences	120	84	36
Number of bacterial OTUs (97% cut off)^a^	422	190	321
Richness estimators^b^			
Chao 1	–	190 (190; 190)	385 (360; 427)
ACE	–	190 (190; 190)	414 (384; 459)
Diversity indices^b^			
Shannon-Wiener	–	3.97 (3.90; 4.04)	4.37 (4.29; 4.46)
Inverse Simpson’s (1/D)	–	18.7 (17.0; 20.7)	15.1 (13.4; 17.4)

^a^ The number of OTU found in the subset of sequences normalized to the sample with the lowest sequence count

^b^ Numbers in parentheses are 95% confidence intervals

The applied 16S rRNA gene amplification protocol was designed for the domain Bacteria. Most of the obtained reads (62%) were assigned to Bacteria (including chloroplast and mitochondrial sequences, which are incorporated into the phylogenetic tree of bacteria due to the endosymbiotic origin of these organelles), however 9.7% of all reads belonged to Archaea. Most archaeal reads were identified in the interior sample (17.8% of total reads) compared to the surface (1.6%), indicating indirectly that Archaea were relatively numerous in the deep layer of the lime. The Archaeal community was dominated by members of genera “*Candidatus Halobonum*” and *Halorubrum*, however it should be noted that the revealed structure could be highly distorted since the applied primers were specific for the domain Bacteria (Klindworth et al. 2013). The estimated number of bacterial species was also much higher in the interior sample, based on both Chao 1 and the Abundance-based Coverage Estimator (ACE) species richness estimators. This value was less than half in the surface sample compared to the deep layer sample. Bacterial diversity was also higher in the deep layer.

**Fig. 3:**
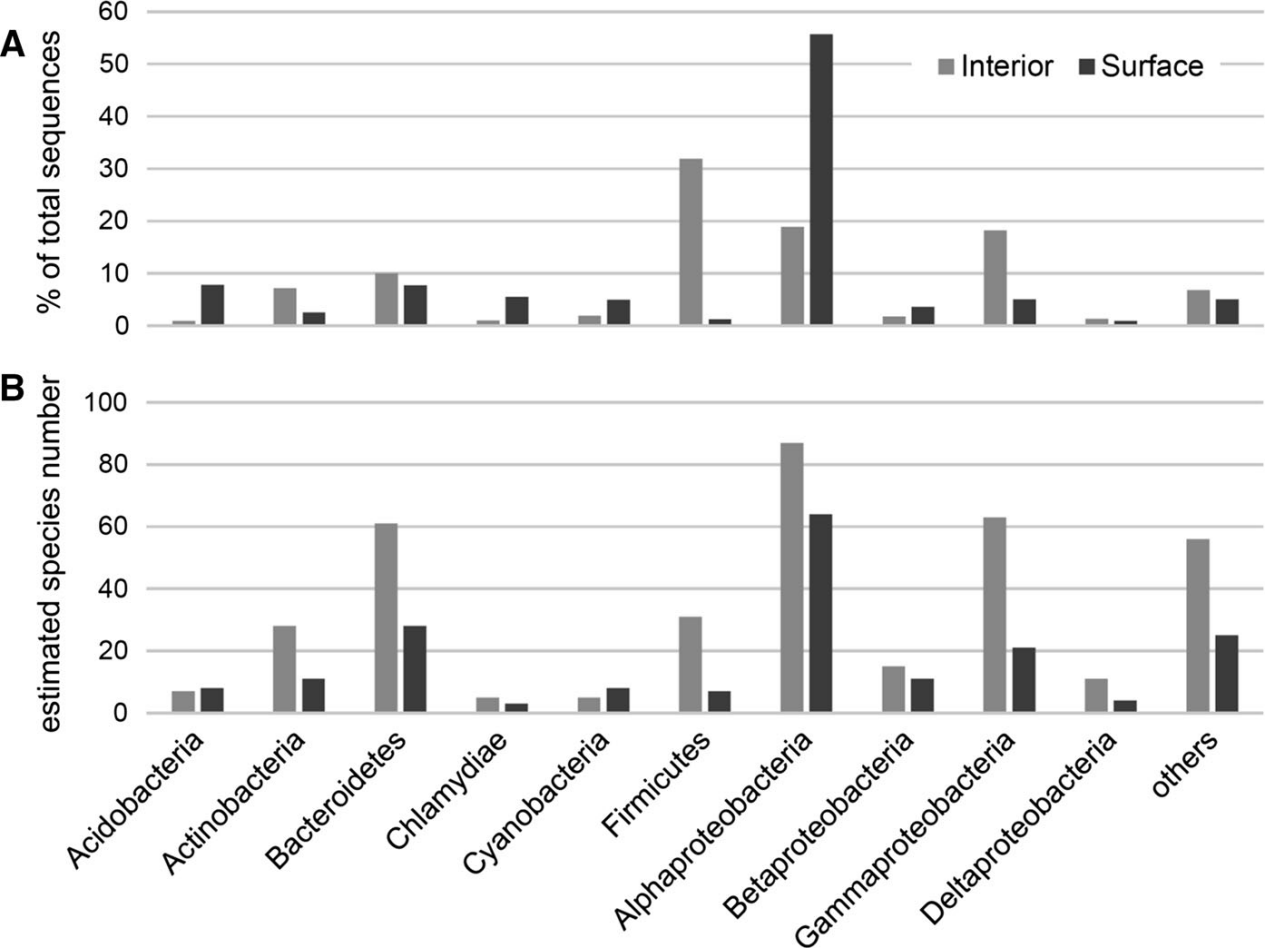
Distribution of bacterial sequences retrieved by pyrosequencing from the interior and the surface saline soda lime samples. Contribution of taxa to the total bacterial community is given based on their relative abundance (**a**) and the distribution of species (**b**). Phylum-level distribution (except for Proteobacteria, where class-level distribution is shown) was expressed as a percentage of total sequences and as estimated species number based on the number of observed OTUs (assigned at 97% similarity level) per group. Species numbers were calculated assuming 97% sequence similarity threshold based on the 16S rRNA gene. Only taxa having contribution higher than 5% at least in one sample are shown

An analysis of the distribution of the most abundant bacterial phyla revealed that the saline soda lime samples were dominated by Proteobacteria, Firmicutes, Bacteroidetes and Actinobacteria (all having >5% contribution to total bacterial reads, excluding chloroplast and mitochondrial 16S rRNA gene sequences; Fig. 3). Proteobacteria was the most abundant phylum in the surface layer of the lime (65.2%) followed by Acidobacteria (7.8%) and Bacteroidetes (7.7%). In the interior sample, 40.3% of the total bacterial sequences belonged to phylum Proteobacteria, followed by Firmicutes (31.9%), Bacteroidetes (10.1%) and Actinobacteria (7.2%). Within the Proteobacteria phylum, the most abundant class was Alphaproteobacteria in the case of the surface sample (representing 85.4% of proteobacterial sequences), while in the deep layer sample, Alphaproteobacteria (46.8%) and Gammaproteobacteria (45.2%) were the most abundant classes.

**Fig. 4:**
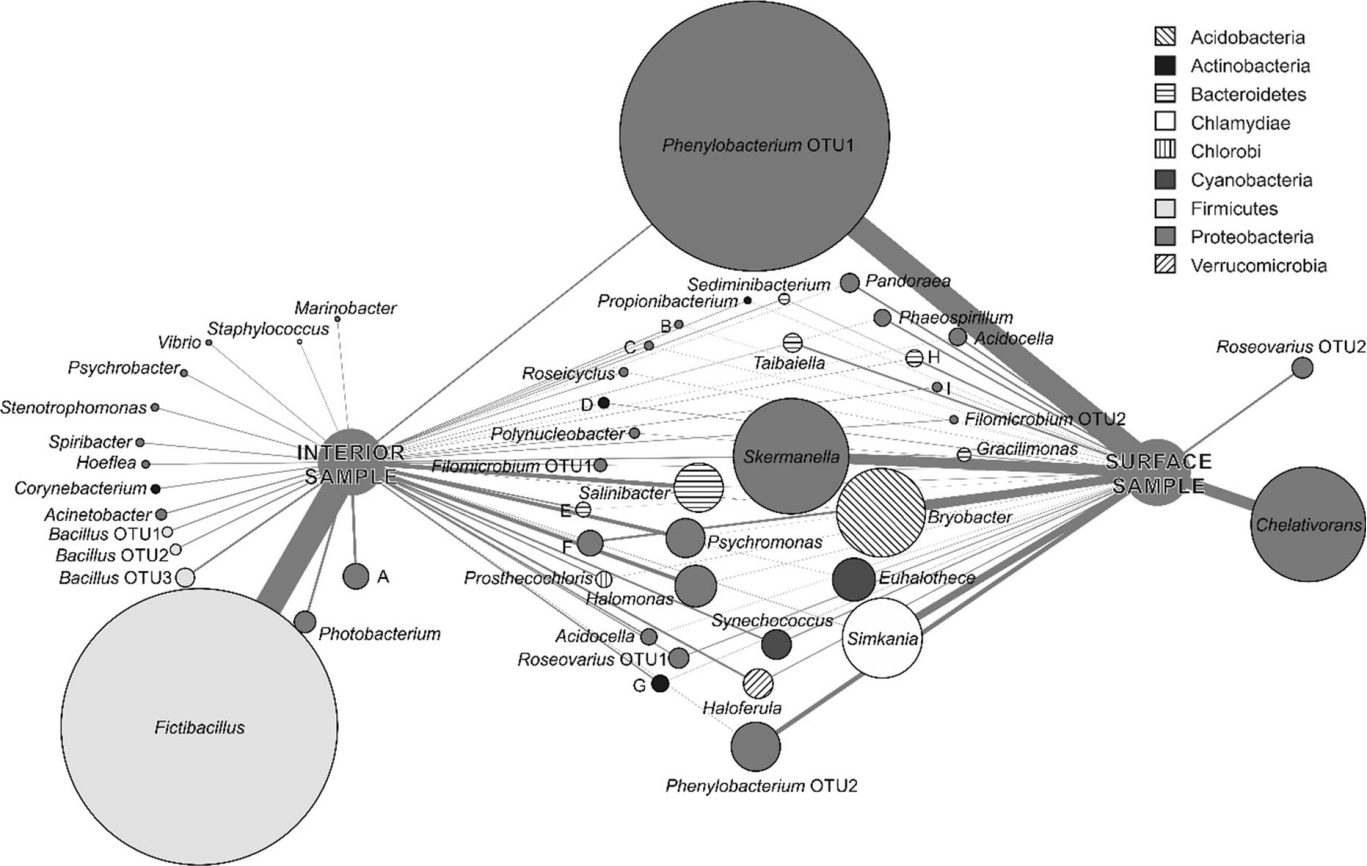
Association network of major bacterial OTUs in the saline soda lime samples based on pyrosequencing data. OTUs were calculated assuming 97% species-level sequence similarity threshold based on the 16S rRNA gene. Top 50 most abundant bacterial OTUs are shown as circles with size corresponding to relative abundance in the total dataset, while the width of the edges corresponds with the relative abundance of an OTU in a particular sample. *Circles* are color-coded according to phylogenetic affiliation and genus-level identity of each OTU is also shown. OTUs distantly related to cultured bacteria were marked with letters as follows: *a* uncultured Rhodobacteraceae, *b* unclassified DB1-14 Alphaproteobacteria, *c* uncultured Rhodobacteraceae, *d* unclassified Elev-16S-976 Actinobacteria, *e* unclassified E6aC02 Sphingobacteriales, *f* unclassified Rhizobiales alphal cluster, *g* unclassified Microbacteriaceae, *h* unclassified ML602 M-17 Bacteriodetes, *i* unclassified NKB5 Gammaproteobacteria

Differences in overall community composition are presented as an association network of major bacterial OTUs (Fig. 4). OTUs belonging to *Phenylobacterium*, *Skermanella*, *Bryobacter, Simkania*, *Salinibacter*, *Psychromonas*, *Halomonas*, *Synechococcus*, *Haloferula* and *Euhalothece* were detected in both layers of the alkaline lime, contributing in different measure to the total bacterial community. On the other hand, the major OTU related to the genus *Chelativorans* was detected exclusively in the surface sample, while *Fictibacillus* and other bacilli (*Bacillus* OTU1-OTU3) were present only in the deep layers of the lime.

**Fig. 5:**
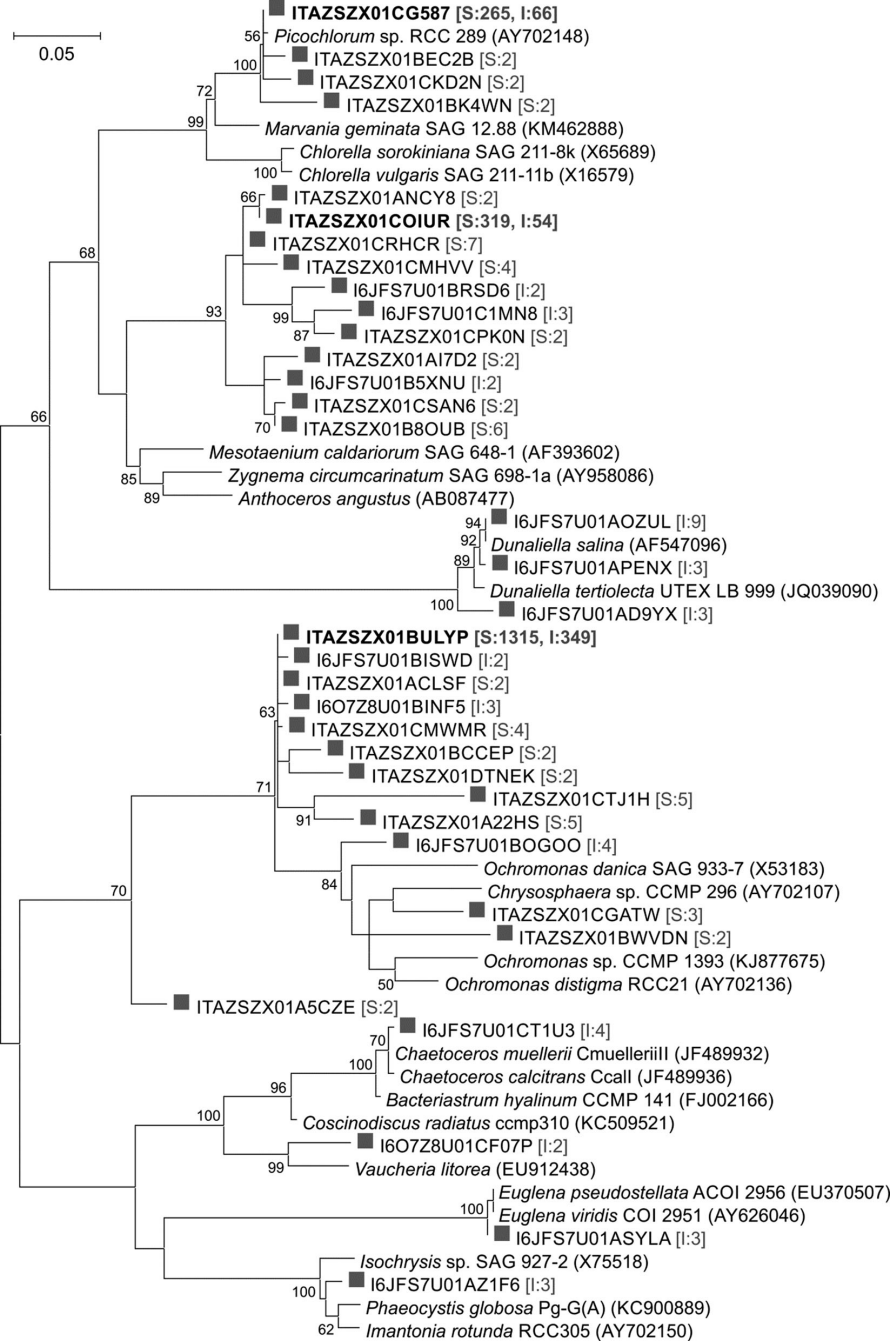
Phylogenetic tree of chloroplast genotypes obtained from the saline soda lime samples based on the pyrosequencing of the 16S rRNA gene. Tree was constructed using the Maximum Likelihood method with the General Time Reversible nucleotide substitution model and is based on 334 nucleotide positions. GenBank accession numbers are given in parentheses. Representative sequences obtained by pyrosequencing are marked with *grey squares*. In this case, sequences differing only in one position are represented with a single sequence, number of sequences is given in square brackets (*I* interior and *S* surface sample) and major phylotypes representing at least 0.5% of total chloroplast sequences are highlighted with *bold letters*

A significant portion of the sequences constituted chloroplasts of algae in both samples (27%, on average). They were more abundant in the surface layer of the saline-alkaline lime (46% of total reads) than in the interior sample (8%). Chloroplast sequences formed three major clades in the phylogenetic tree presented in Fig. 5. The most abundant were reads related to the genus *Ochromonas* (Chrysophyceae, Stramenopiles; representing 68.9% of the total number of chloroplast reads), while the others were related to the genera *Mesotaenium* (Zygnemophyceae, Streptophyta; 14.8%) and *Picochlorum* (Trebouxiophyceae, Chlorophyta; 13.4%). Other detected genera included *Dunaliella* (Chlorophyceae, Chlorophyta), *Chaetoceros* (Bacillariophyta) and *Euglena* (Euglenozoa).

### Bacterial isolates

**Fig. 6:**
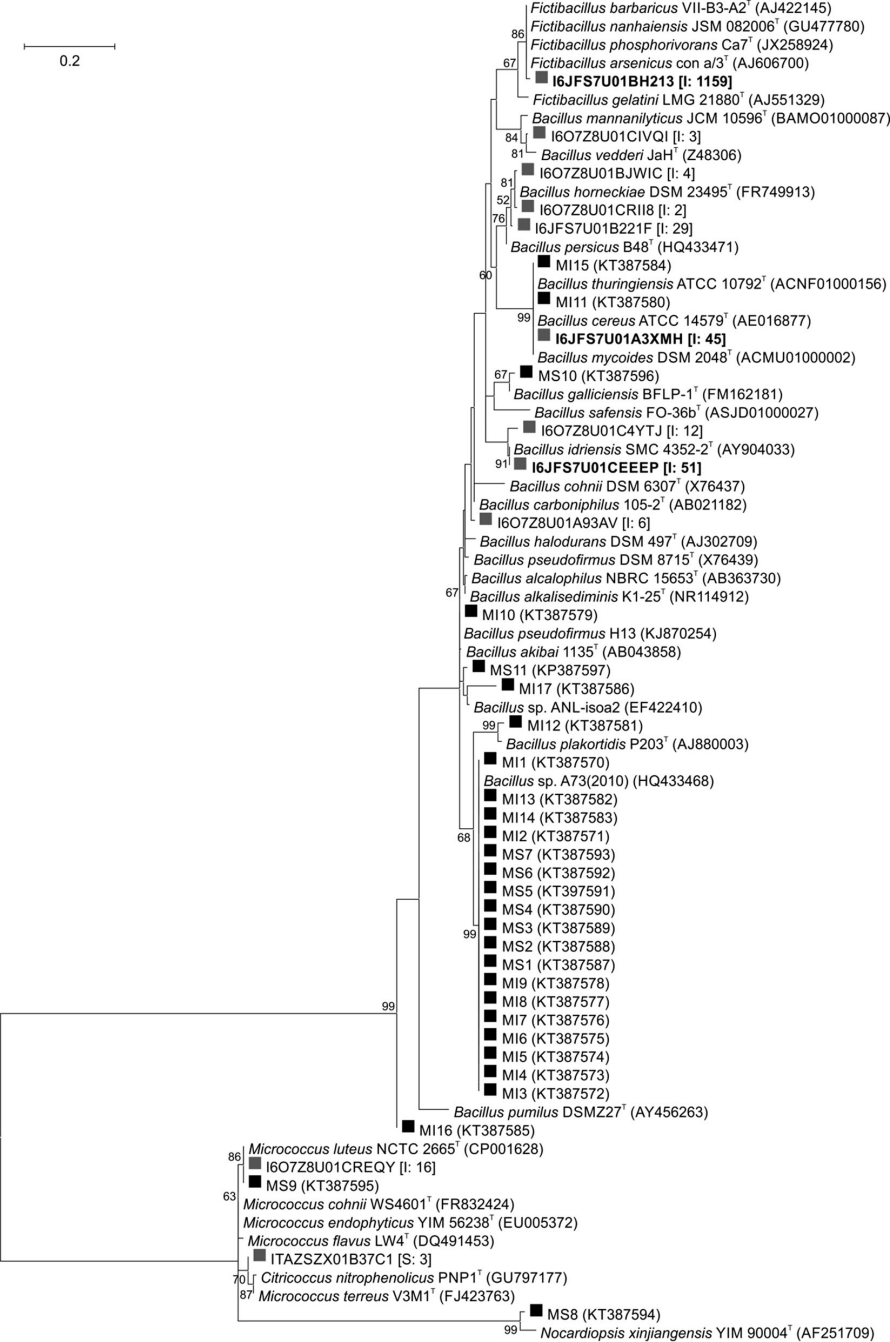
Phylogenetic tree of the saline soda lime bacterial isolates and closely related genotypes from pyrosequencing data based on the 16S rRNA gene. Tree was constructed using the Maximum Likelihood method with the Kimura 2-Parameter nucleotide substitution model and is based on 356 nucleotide positions. Type strains are marked with superscript T, GenBank accession numbers are given in parentheses. Bacterial isolates are marked with *black squares*, while representative sequences obtained by pyrosequencing are marked with *grey squares*. In the latter case, sequences differing only in one position are represented with a single sequence, number of sequences is given in square brackets (I, interior and S, surface sample) and major phylotypes representing at least 0.5% of total bacterial sequences are highlighted with *bold letters*

Eleven isolates were retrieved from the surface (marked MS, strains MS1–MS11) and seventeen from the interior sample (marked MI, strains MI1–MI17). All of the bacterial isolates stained Gram-positive. Molecular taxonomic identification of the isolates (Fig. 6) revealed a very low diversity level of culturable bacteria. The strains belonged to three genera: *Bacillus, Micrococcus* (one strain), and *Nocardiopsis* (one strain). The closest relative of 18 strains (MS1–7, MI1–9, MI13, MI14) was *Bacillus* sp. A73, first isolated from a hypersaline lake in Iran (Amoozegar et al. 2014). Strain MI12 was identified as alkalitolerant *Bacillus plakortidis*, which was first isolated from a sponge in the Norwegian Sea (Borchert et al. 2007). The closest relative of isolate MI17 was *Bacillus* sp. ANL-isoa2 and its isolation source was a solonchak soda soil in the Kulunda Steppe, Mongolia (Sorokin et al. 2007). The closest relative of isolate MI10 was a moderately halotolerant and obligately alkaliphilic *Bacillus pseudofirmus* strain H13, isolated from the lime and described previously (Kalwasińska et al. 2015). *Bacillus cereus* was the closest relative of strain MI11, and *Bacillus thuringiensis* was the closest relative of strain MI15; however, it is not possible to distinguish between these two species on the basis of gene sequences that are not related to the production of entomopathogenic toxins (Sorokin et al. 2006). Isolate MI16 was identified as *Bacillus pumilus*, another alkalitolerant and common soil species. The closest relative of strain MS8 was *Nocardiopsis xinjangensis*, a halophilic actinomycete isolated from a saline soil in China (Li et al. 2003) and the closest relative of strain MS9 was *Micrococcus luteus*, which is ubiquitous in nature.

## Discussion

The saline soda lime is an unusual environment created by humans as a result of the chemical processes of soda production. This extreme environment, consisting almost entirely of calcium carbonate, is highly alkaline, saline and nutrient-poor, especially in terms of nitrogen and organic carbon content. Most of the organic matter present in this artificial environment originates from allochtonous airborne material and atmospheric deposition on the lime surface. A comparison of the bacterial diversity observed in this study with that of other studies is difficult because of the lack of papers describing similar environments.

The high alkalinity caused by the presence of carbonates, bicarbonates and hydroxides, as well as the high concentration of salts, are thought to be the key factors that shape the structure of microbial communities in this environment. The influence of pH and salinity on bacterial and archaeal community composition in various ecosystems was extensively investigated by other researchers (Liu et al. 2015; Wu et al. 2017; Maturrano et al. 2006; Zhang et al. 2013; Canfora et al. 2014). These studies have definitively shown that pH and salinity are the most important factors controlling microbial abundance, metabolic activity, and diversity.

The total number of bacteria in the lime (10^6^–10^8^ cells g^−1^) was low compared to dolomites and soil, where it ranged from 10^8^ to 10^9^ cells g^−1^ (Stres 2007; Sheibani et al. 2013). This may be connected with the very harsh physicochemical conditions mentioned above. In general, a more extreme environment is expected to maintain a lower abundance of microorganisms by selecting a small group of highly specialized organisms able to survive and grow under such demanding conditions (Rothschild and Mancinelli 2001). On the other hand, the observed bacterial counts were quite similar to those obtained for highly alkaline and saline Mono Lake (Humayoun et al. 2003) or cave environments made of limestone (Barton and Jurado 2007; Wu et al. 2015).

The higher quantity of culturable bacteria and the total cell counts, as well as the higher community species richness and the diversity detected in the interior, compared to the surface layer of the saline soda lime, may be related to the more stable environmental conditions existing in the deeper layer of the lime. The flat, vegetation-free, semisolid surface layer of the lime is subjected to periodic fluctuations in moisture and temperature, and as a consequence, in salinity and pH. Due to the high evaporation rate and low precipitation rate (below 500 mm year^−1^) in the Kuyavia region, Central Poland (Kędziora 2011), the studied area is similar to cold, semi-arid habitats. Microorganisms in this environment must adapt to changing environmental conditions such as long dry periods during late spring and summer. Desiccation cracking of the surface layer of the lime can substantially reduce microbial activity and growth (Pesaro et al. 2004) and change the microbial community structure (Thion and Prosser 2014). Furthermore, microbial biomass and activity in the lime may decrease with the increasing number of drying and rewetting cycles, as is observed in soils (Mikha et al. 2005; Wu and Brookes 2005).

Due to the high alkalinity connected with the presence of Ca(OH)_2_, the saline soda lime increases the pH of aqueous solutions up to 11–12 within a few minutes (Jeliński et al. 2011). The repeated shifts between moderate and highly alkaline conditions, as well as the presence of drying and rewetting cycles, make the surface of the lime more changeable and selective for microorganisms than the deeper layers of the lime. A similar increase in diversity in the interior layer, compared to the surface, was observed in other extreme, alkaline environments like submarine ikaite columns (made of calcium carbonate hexahydrate, low salinity) located in Greenland (Glaring et al. 2015) and in alkaline-saline Mono Lake, California, and Soap Lake, Washington, when going down from the light-exposed, oxic surface waters to the anoxic bottom (Humayoun et al. 2003; Dimitriu et al. 2008).

The distribution of the most abundant bacterial phyla in the alkaline, saline soda lime was similar to that observed for submarine ikaite columns (Glaring et al. 2015) and saline desert Kutch (Pandit et al. 2014) and slightly different than that found in soda lakes, however that may be due to the different methodology applied. Most of the previous studies of solonchaks or soda lakes have been based on the construction of 16S rDNA clone libraries and subsequent sequencing of individual 16S rDNA clones. Since only limited amounts of clones were sequenced, the complexity of the intrinsic community could not be revealed. Pyrosequencing technology provides a large number of sequence reads in a single run, resulting in deep datasets and allowing for the detection not only the most abundant microbial community members but also the low abundance taxa (Pershina et al. 2012; Dudhagara et al. 2015). Nevertheless, it should be mentioned that pyrosequencing of 16S rRNA amplicons does not imply that the taxa found were actively growing in the saline soda lime, since the detected DNA could be derived from dormant or dead microorganisms.

The most abundant heterotrophic bacterial OTU present in the saline soda lime belonged to the genus *Phenylobacterium* (Alphaproteobacteria) and it was dominant in the surface layer. Members of this genus are strict aerobes or facultative anaerobes and have been isolated from various environments, including highly alkaline, oligotrophic groundwater (Tiago et al. 2005). However, thus far there has been no evidence of their presence in other caustic, saline, or lime habitats. Some members of the genus *Phenylobacterium* are xenobiotic compound-degrading bacteria, and some of them can reduce nitrate to nitrite (Oh and Roh 2012). Others have a unique adaptation that allows them to thrive in oligotrophic environments (Abraham et al. 2008). This last feature of the representatives of the genus *Phenylobacterium* is certainly important in terms of their abundance in the nutrient-poor lime under study.

Autochthonous microorganisms can survive in extreme environment owing to their ability to form spores. The second most abundant bacterial OTU in the lime belonged to the genus *Fictibacillus* (phylum Firmicutes), which contains species recently reclassified from the genus *Bacillus* (Glaeser et al. 2013). Representatives of the phylum Firmicutes were also relatively abundant in metagenomes from saline and alkaline desert Kutch, India (Pandit et al. 2014), a saline and alkaline Mojave Desert playa lake (Navarro et al. 2009), and sediments of hyper saline and hyper alkaline Lonar Lake (Paul et al. 2016) and much less abundant in a limestone cave environment (Wu et al. 2015). One of the *Fictibacillus* representatives, halotolerant and alkaliphilic *Fictibacillus solisalsi*, was isolated from saline soil (Liu et al. 2009) resembling soda lime in terms of salinity and alkalinity. Surprisingly, in the soda lime, *Fictibacillus* was detected only in the interior samples. Nevertheless, alkaliphilic spore-forming Bacilli show a worldwide distribution and are likely to be spread widely by the wind and migratory birds (Jones and Grant 2000).

Representatives of *Chelativorans* (order Rhizobiales) and *Skermanella* (order Rhodospirillales) within the class Alphaproteobacteria were also among the abundant OTUs found in the surface layer of the lime. These Gram-negative, strictly aerobic bacteria were previously isolated from various environments, including nutrient-poor environments (air, desert soil, and coal mine soil (Weon et al. 2007; An et al. 2009; Luo et al. 2012). However there is no evidence of their presence in alkaline environments like soda lakes or limestone.

Members of phylum Acidobacteria were also present in the saline soda lime, especially in its surface layer. This is not surprising since Acidobacteria is the second most abundant taxon in some soils (Janssen 2006) and its representatives may be easily spread by wind. This group of bacteria is known to be more abundant in environments with low carbon availability (Fierer et al. 2007) and many members of Acidobacteria have been found to be abundant in alkaline soils (Dunbar et al. 2002; Rousk et al. 2010). The detected genus *Bryobacter* includes a type strain that was first isolated from Sphagnum-dominated acidic wetlands (Kulichevskaya et al. 2010), suggesting the presence of micro-habitats in the lime matrix that allow organisms sensitive to high pH to survive in this alkaline environment.

Members of the genus *Halomonas* (Gammaproteobacteria), encompassing moderately halophilic and halotolerant bacteria (Wang et al. 2014), were detected in the saline, alkaline soda lime, especially in the interior layer. Their presence in the lime is fully justified, as they show widespread distribution in saline and also alkaline environments like solonchaks and soda lakes (Shapovalova et al. 2009; Duckworth et al. 2000).

The most surprising is the presence of the genus *Simkania* (phylum Chlamydiae) in the surface layer of the highly alkaline and saline lime. Its representatives are obligate intracellular pathogens (Michel et al. 2005), however it is difficult to identify their host in this extremely harsh environment.

Hypersaline environments are generally dominated by halophilic Archaea, which belong to the order Halobacteriales (Ventosa and Nieto 1995). Since the applied PCR primers were designed to amplify members of the domain Bacteria, archaeal sequences obtained by pyrosequencing may present a highly distorted community structure. However, archaeal reads belonging to “*Candidatus Halobonum*” and *Halorubrum* (Halobacteriaceae) were still conspicuously present in the saline, alkaline lime deposit, especially in its deep layer.

Apart from Archaea, the bacterial genus *Salinibacter*, an extremely halophilic taxon within the Bacteroidetes with archaeal-like properties was detected in the saline, alkaline soda lime, especially in the interior layer. *Salinibacter* shares many features with the family Halobacteriaceae: both groups use KCl for osmotic adjustment of their cytoplasm, both mainly possess salt-requiring enzymes with a large excess of acidic amino acids, and both contain different retinal pigments (Oren 2013).

Primary producers present in the alkaline, saline lime were both prokaryotic cyanobacteria and eukaryotic algae. Cyanobacteria are among the most alkaliphilic microbes, and they frequently dominate alkaline environments, such as soda lakes and microbial mats (Somogy et al. 2010; Dadheech et al. 2013). They also constitute an important group of phototrophs in saline desert soils (Li et al. 2013; Pandtit et al. 2014). Therefore, their presence in the alkaline and saline surface layer of the soda lime can be expected. In particular, *Euhalothece* and *Synechococcus* phylotypes, detected in the lime, are among the most prominent inhabitants of hypersaline and alkaline soda environments (Garcia-Pichel et al. 1998; Feloföldi et al. 2011).

Most of the chloroplast sequences in the surface layer of the lime were distantly related (<93% pairwise sequence similarity) to the genera *Ochromonas* (Chrysophyceae) and *Mesotaenium* (*Zygnemophyceae*). Therefore, eukaryotic algal phylotypes detected in the studied samples may represent novel phylotypes, and most probably belong to genera which have yet to be described. Picoeukaryotic and phototrophic members of the genus *Picochlorum*, which carry sequences that were found in the saline soda lime, were also reported in various saline environments, both marine (Henley et al. 2004) and continental (Máthé et al. 2014). This is probably due to the broad halotolerance of some *Picochlorum* representatives (e.g. *P. oklahomense*, Henley et al. 2002). Plastid sequences related to *Dunaliella* were also found in the lime and this is consistent with common distribution of this genus in saline environments, such as the Dead Sea, Israel, solar salterns (Oren 2010) and the Great Salt Plains, USA (Buchheim et al. 2010).

The number of culturable, aerobic bacteria grown on an alkaline medium (10^3^–10^4^ CFU g^−1^ dry mas) was within the range observed for weathered limestone (Mulec et al. 2001), soils (Horikoshi 1991), soda lakes (Joshi et al. 2008) and salterns (Maturrano et al. 2006). However, it was lower than in alkaline alpeorujo (10^5^ CFU g^−1^), an organic sludge-like by-product of olive oil extraction (Ntougias et al. 2006) probably due to the lower amount of nutrients present in the lime and its high salinity. The low content of organic carbon and nitrogen are factors significantly limiting the growth of any heterotrophic community of microbes.

Most of the bacteria isolated from the alkaline and saline lime were alkaliphiles and moderate halophiles. Molecular phylogenetic identification of the strains revealed that culturable diversity was very low compared to other alkaline and saline environments (Antony et al. 2013; Grant and Jones 2016) since members of only three different genera, namely *Bacillus*, *Nocardiopsis,* and *Micrococcus*, were retrieved. A previous study of this environment, focusing on alkaline lime ponds of lower salinity, revealed the presence of other culturable genera: *Halomonas*, *Planococcus*, and *Microcella* (Kalwasińska et al. 2015). Nevertheless, the observed diversity of isolates from alkaline lime was still low and may reflect an inability to create the optimal growth conditions for microorganisms derived from an extreme habitat in the laboratory. On the other hand, the observations performed during the present investigation remain consistent with those of other researchers. Reported species, especially *Bacillus*, *Halomonas*, and *Micrococcus* are described to be alkali- and halotolerant and they exhibit a broad environmental distribution, therefore their common occurrence in alkaline and saline artificial lime is to be expected (Kamekura 2013; Grant and Jones 2016).

## Conclusions

The data presented shows that surprisingly diverse, distinct bacterial communities are present in the highly alkaline, saline and nutrient-poor environment of soda lime (Poland). The examined artificial environment maintains extremophilic bacterial taxa adapted to thrive in this unusual ecosystem. Most of the detected bacterial and plastid sequences were related to microorganisms found in various alkaline and saline, nutrient-poor environments such as solonchaks, desert soils, limestone, soda lakes and ponds, oceans, saline springs and alkaline ground water or even air. The phylogenetic groups identified in the surface and in the interior layers of the lime were distinct. Higher bacterial abundance and diversity was detected in the deep zone of the lime than in the surface layer, possibly due to both geochemical and temporal stability differences between these two environments. Molecular phylogenetic identification of the isolates revealed a very low diversity of culturable bacteria, which belonged mainly to the genus *Bacillus*, which is widely distributed in different saline and alkaline habitats with high pH and a variable salt concentrations.

## Electronic supplementary material

Below is the link to the electronic supplementary material.

Supplementary material 1 (DOCX 16 kb)
