# Supplementary material for: Microbial communities associated with the anthropogenic, highly alkaline environment of a saline soda lime, Poland
Source: Antonie Van Leeuwenhoek. 2017 Apr 5;110(7):945–62. doi: 10.1007/s10482-017-0866-y (PMC5486852; doi:10.1007/s10482-017-0866-y)
Supplement: Supplementary file 1 — Supplementary material 1 (DOCX 16 kb) [file 10482_2017_866_MOESM1_ESM.docx]

**Fig. S1** Rarefaction analysis of sequences from alkaline soda lime

Rarefaction curves of bacterial pyrosequencing data applying 97% similarity threshold of the 16S rRNA gene
